# Models of religion-related education in Germany, Switzerland and Austria and the topic of Islam

**DOI:** 10.1080/13617672.2025.2488709

**Published:** 2025-05-20

**Authors:** Benjamin Ahme, Sabine Hermisson

**Affiliations:** a Faculty of Theology, Humboldt-University Berlin, Berlin, Germany; b Department of Religious Education, Protestant-Theological Faculty, University of Vienna, Vienna, Austria

**Keywords:** Regionality, comparative RE, models of RE, Islam

## Abstract

Regionality as a formative characteristic of religion-related education in Germany, Switzerland and Austria is the focus of the current project ‘Models of Religious Education and the topic of Islam’ (‘MORE’). The project compares four different regional variants of religion-related education that are assigned to two competing models, denominational RE and non-denominational RE, by investigating how each of them presents the topic of ‘Islam’. The comparison is carried out on the basis of qualitative research through interviews with teachers and pupils as well as lesson observations. Comparing regional variants of RE calls for a study design that is, on the one hand, attentive to regional characteristics while also reaching a degree of standardisation that allows for comparisons, on the other hand. Following trends in comparative education, the article describes the architecture of the MORE project as a type of collaborative cross-border research.

## Regionality in religion-related education in German-speaking countries

Regionality is a formative characteristic of religion-related education in the three German-speaking countries Germany, Switzerland and Austria which are considered in this article. Education in general – and religion-related education in particular – varies distinctly both among these three countries, but, especially with regard to Germany and Switzerland, also among individual regions. This is one of the reasons which make studying the situation in these countries especially worthwhile when the question of regionality in religion-related education^1^ comes up.

The aim of this article is to show how regionality has been included in the design of a large international, comparative and empirical study that investigates differences in the treatment of the topic ‘Islam’ between competing models of RE in four different regions. The current project ‘Models of RE and the topic of “Islam”’ (MORE), on which this article is based, has its focus on different models of RE in their regional variation in Germany, Switzerland and Austria.^2^ Regionality is understood as a subnational point of reference that enables differentiations within nation states according to contextual factors such as policy, culture or historical developments. In the first section of the article, it will be explored how regional contexts affect the teaching of the topic of ‘Islam’ in German-speaking countries. The second section then explains how regionality has been included in the design of the study.

## MORE: four regional variants and two models of RE

Regionality in German-speaking education is due to two factors:

1. *Educational pluralism*: Both in Germany and Switzerland the responsibility for the schools lies with the individual federal states, in Germany with the 16 Länder, in Switzerland with the 26 Kantone, of which 17 are German-speaking. The federal states are responsible for the individual school system (types of schools, range of subjects, etc.), for the training and employment of teachers as well as the syllabi for the respective subject. This results in considerable differences between regions which affect all subjects.
2. *Historical developments*: Throughout the centuries, historical developments have shaped the religious landscapes of this trinational area. The Reformation diversified the religious situation (Luther, Zwingli, Calvin) and brought forth a mosaic of religious traditions. More recently, the rise and fall of the GDR has left a strong secular legacy in Eastern German. Especially in the last two decades, different patterns of migration have resulted in an increase in religious plurality, in particular, in urban areas such as Zurich and Vienna. These developments have a strong impact on RE.

As a result of both educational pluralism and historical developments, the trinational area of Germany, Switzerland and Austria features a spectrum of regional variants of RE. They are often roughly assigned to two competing models, denominational RE and non-denominational RE.

### Four regions and their variants of RE

The MORE project involves four regions which present four distinct variants of RE: Greater Stuttgart area/Baden-Württemberg (southwest Germany), Greater Potsdam area/Brandenburg (eastern Germany), Greater Zurich area (Switzerland) and Greater Vienna area (Austria). In Baden-Württemberg and Austria, where RE classes are taught separately according to denominations and religions, MORE focuses on Protestant RE in order to do justice to the particularities of these regions. The following subsections will outline how contextual factors from levels such as national and federal legislation, religious history and migration lead to different conditions for teaching the topic of ‘Islam’ in the four regions.

#### Greater Stuttgart area/Baden-Württemberg (Southwest Germany)

The state of Baden-Württemberg has a tradition of high rates of church membership, divided in equal parts between Protestants and Roman Catholics. While church membership decreased since the 1960s, and the religious landscape diversified due to immigration, with a growing percentage of Muslims (7% to date), still about 60% of the population are church members with the share of Protestants and Roman Catholics about equal (Bundeszentrale für politische Bildung 2020).

In Germany’s basic law, RE is conceptualised as a so-called *res mixta* – a joint matter of state and religious communities (such as the Protestant Church in Germany and its Catholic – but also Muslim etc. – equivalents), with the state providing for the organisational framework and religious communities defining teaching objects and curricula. In most federal states (12 out of 16, including Baden-Württemberg) this translates into denominational RE which is predominantly Protestant RE and Catholic RE but includes, in principle, also other denominations and religions such as Islamic RE, Jewish RE, etc. (Meyer-Blanck 2014).

‘Protestant RE’ means Protestant RE teachers and curricula defined by the Protestant Church. With respect to pupils, Protestant RE is open for all pupils, regardless of their denomination or religion. In practice, pupils are predominantly Protestant, but classes often include non-affiliated pupils and occasionally pupils with other religious affiliations, especially in elementary schools where an alternative subject is usually missing. Pupils can opt out of RE. Where the alternative subject ‘Ethics’ is implemented, attendance of either RE or the alternative subject ‘Ethics’ is mandatory (Boschki and Schweitzer 2020).

This means that for Protestant RE in the greater Stuttgart area, the topic of ‘Islam’ is usually taught in groups of 15–25 pupils that occasionally include Muslims but mostly consist of Protestants. For each year level, the curricula feature sections on ‘Religions and Worldviews’ other than Christianity and aim at ‘conveying basics for interreligious contacts’ (Baden-Württemberg 2016).

#### Greater Vienna area (Austria)

Austria used to be a Roman Catholic country with high membership rates for the Roman Catholic Church of around 90% of the population. With church membership declining in the last decades, today about 50% of the Austrian population are members of the Roman Catholic church. In contrast, Protestants are a very small minority, with only about 4% of the population – half of the percentage of Muslims (8%) which have increased considerably in the last decades.

By and large, Austria implements the same model of RE as Baden-Württemberg, with the state providing for the organisational framework and religious communities defining teaching objectives and curricula. In practice, this is predominantly Catholic RE, but at many schools, there also is Protestant and Islamic RE. In principle, RE can, however, also be taught by other denominations and religions such as Orthodox, Jewish or Buddhist.

The fact that Protestants are such a small minority has a considerable impact on the reality of Protestant RE in Austria. It entails small numbers of Protestant pupils at most schools, often tiny RE classes with sometimes as few as three pupils (occasionally across year-groups and across schools), and RE teachers who usually teach in several schools. While Catholicism continues to dominate, there is legal protection for minorities with regard to RE. This is expressed, for example, in the fact that pupils are only allowed to attend the RE of their respective religious affiliations and that minorities are permitted to organise their RE across schools (Rothgangel 2024).

Protestant RE in Austria means Protestant RE teachers, curricula defined by the Protestant church, and predominantly Protestant pupils, plus often some unaffiliated pupils. Unlike in Baden-Württemberg there has long been no mandatory alternative subject – another reason for RE attendance to decrease as those pupils who do not attend RE have a free period or are off from school early. However, in 2021, ‘Ethics’ has been introduced as a mandatory alternative subject for grades 9–12. Despite the similarities regarding the general model of RE in the greater Stuttgart and greater Vienna area, RE classes in which the topic of ‘Islam’ is taught, differ considerably. This also includes the teachers, who in Vienna often only teach RE – but at multiple schools – whereas in Stuttgart, they usually teach at one school and also cover a second subject.

#### Greater Potsdam/Brandenburg (Eastern Germany)

In the eastern part of Germany, historically a predominantly Protestant region, the anti-church policy of the GDR regime has left a deep mark with widespread secularisation and the diaspora and minority status of churches. In Brandenburg in 2023, church membership was down to 18% of the population (Bundeszentrale für politische Bildung 2020). The percentage of Muslims is 0.8%, the second smallest proportion of the population compared to the other German states.

After the fall of the GDR, the federal state of Brandenburg was investigating new ways of organising the school system, including RE that would be in line with the transformation of society as a whole and the deeply secular legacy of the GDR. In a multi-phased process, the subject ‘Lifestyle-Ethics-Religious Studies’ (L-E-R)^3^ was established in the late 1990s. It was designed programmatically as a non-denominational subject that is explicitly neutral with regard to religion and worldviews. The term ‘Religious Studies’ – instead of ‘Religion’ – was intended to reflect the difference to denominational RE which was regarded as not fitting for the Brandenburg context. Since 2008, L-E-R has been taught across the entire state in grades 5 to 10 (Lenz 2020).

L-E-R is unique in Germany, where denominational RE prevails. It is mandatory for all pupils (although at least legally, they can opt out under certain presuppositions) and is guided by the objective of introducing the pupils to the phenomenon of religion and world religions (R) and philosophical ethics (E) within the context of topics which the pupils encounter in their lifeworld (L) (University Potsdam 2025). This compound nature of the subject means that the topic of ‘Islam’ should, in principle, not be taught on its own, but as a recurring dimension of themes derived from the pupils’ lifeworld.

#### Greater Zurich area (Switzerland)

As in Germany and Austria, also in Switzerland, church membership has declined during the last decades from over 90% in 1970 to currently around 60% of the population with Catholics as the largest denomination, followed by the Reformed denomination (Bundesamt für Statistik 2025). In the same period, the proportion of the population without religious affiliation increased drastically from 1% to 33%. In Zurich, according to the Swiss Federal Statistical Office, more than one-third of the population have no religious affiliation in 2021. The proportion of Muslims has increased to 7%.

Due to this considerable pluralisation of religious affiliation or non-affiliation, in 2005 in the Canton of Zurich denominational RE was replaced by a non-denominational subject under the title ‘Religion and Culture’. With the introduction of the so-called Curriculum 21, ‘Religion and Culture’ was supplemented with ethics and is called ‘Religions, Cultures, Ethics’ (RKE) since 2018. Attendance is mandatory for all pupils.

For RKE, the regulations emphasise religion to be taught in the sense of teaching about religions, not instruction in a religion with the latter regarded as a matter for parents, churches and faith communities. The objective is for pupils to develop skills for living with different cultures, religions, worldviews and values (Schlag 2023). As in L-E-R, RKE integrates religion into a wider interdisciplinary framework. Especially in the regional context of Zurich, though, this takes place in a religiously much more plural setting with more Muslims than in the greater Potsdam area.

### The discussion on denominational vs. non-denominational RE

Both Baden-Württemberg and Vienna implement the denominational model of RE and, consequently, share a number of common features: RE (in the case of Protestant RE) is taught (1) according to teaching objects and curricula provided for by the Protestant Church, (2) by Protestant teachers to (3) often predominately Protestant pupils.

In contrast, Greater Zurich and Potsdam/Brandenburg both implement the non-denominational model of RE: RE is taught (1) according to teaching objects and curricula provided for by the state, (2) by teachers without mandatory religious affiliation – and often to explicitly non-affiliated or even explicitly non-religious - (3) pupils of all kinds of religious and non-religious backgrounds.

In RE research, denominational RE and non-denominational RE are often regarded as two competing models and are, at times, the objects of heated debates (e.g. Franken and Loobuyck 2011; Gärtner 2015; Kenngott, Englert, and Knauth 2015; Lindner et al. 2017; Riegel 2018; Schröder 2014). Denominational RE is often assumed to be one-sided (e.g. Alberts 2023), because it proceeds from a particular religious perspective. In contrast, non-denominational RE is often criticised for insufficiently considering the substantial core of religious traditions and the aspect of personal commitment related to religion (Schlag 2021).

However, there are two points to be considered that have so far been largely neglected in this debate:

1. *Classroom-level research*: The claims concerning differences between the two models of RE and the advantages of one model over the other are hardly ever based on empirical research. There is not much empirical evidence on whether the differences are reflected in the reality of teaching RE.
2. *The formative effect of regionality versus model*: As seen above, the contextual differences between two manifestations of the same model can be considerable.

While both Greater Stuttgart/Baden-Württemberg and Vienna/Austria implement the denominational model, the practice differs markedly: learning group with normal class size drawn from two or three classes in Baden Württemberg versus mostly tiny RE classes, occasionally across year-groups and even across schools, in Austria.

This applies accordingly to Greater Zurich and Potsdam/Brandenburg who both implement the non-denominational model of RE but differ considerably due to the increasing plurality of religious and non-religious affiliations in Zurich versus a widespread secularisation as heritage of the former GDR. Furthermore, in these cases RE is integrated into compound subjects that also address matters of culture, ethics or lifeworld and that place additional demands on teaching the topic of ‘Islam’. Why this topic is suitable for a comparison of different models of RE in different regions will be addressed in the next section.

## Research focus: the topic of ‘Islam’ and comparative research design

This section will focus more closely on how a comparison between the regions and models described above can be carried out. The overarching research question that the MORE project is exploring is as follows: How is the topic of ‘Islam’ presented by teachers and experienced by pupils in the four regional variants and two models of RE? This focus involves a spectrum of nuanced questions regarding the four locations and two models, e.g.: Is it possible to identify differences in the teachers’ motivations, views, competencies, aims and didactic designs? Is it possible to identify differences in the pupils’ views and experiences? The aim is to derive more nuanced insights into the formative effects of the regional context and the respective models of RE based on empirical findings.

### The topic of ‘Islam’ as an indicator

As RE curricula consist of many features that cannot all be included in a comparison, the MORE project employs an indicator-based approach to explore commonalities and differences between the four regional variants of RE and to investigate the formative effects of regionality and model. The way of teaching the topic of ‘Islam’ was selected as such an indicator.

In all four regions, ‘Islam’ is taught in RE at secondary school level, which makes it a suitable indicator because it offers a shared point of reference. The topic of ‘Islam’ is also of particular interest, as it is the religion that most people feel affiliated with after Christianity in Germany, Switzerland and Austria, albeit at a much lower percentage of the population. Tensions regarding the relation between Christianity and Islam and concerning Islam and society in general are often blamed for social conflicts, prejudice and hostility. In particular, against the backdrop of the debate on denominational RE versus non-denominational RE, the topic of ‘Islam’ can be expected to bring to light differences between denominational and non-denominational RE – if they really do exist. This could include, for example, denominational RE teachers presenting a recognisable Christian view on Islam – maybe with a strong emphasis on ‘us’ versus ‘them’ or non-denominational teachers employing a world-religions approach.

### Qualitative data and regionality in comparative methodology

A comparison between the regional RE variants is carried out based on qualitative research and the possibility of meaningful comparisons rests on the quality of the underlying data. To achieve a differentiated picture, the empirical approach integrates different perspectives on how the topic of ‘Islam’ is taught. At each of the four regions, interviews were conducted with teachers and groups of their pupils. In addition, lessons in which the topic of ‘Islam’ was taught by the interviewed teachers and that were attended by the interviewed pupils were observed. Besides contextual differences, this approach implies challenges due to the breadth and the varied nature of the data to be analysed and interpreted. These challenges and how they are integrated into the study design will be addressed in the next subsection as a contribution to the methodological discussions in comparative RE. Integrating such a methodological perspective into the project is not only an end in itself for developing the design of the MORE project but also a reaction to a general lack of methodological literature concerning international comparative qualitative studies especially in the field of RE. Large international studies such as Rel-Edu (Rothgangel, Jäggle, and Schlag 2016) or TRES (Ziebertz and Riegel 2009) have mostly focused on aspects other than the design of qualitative studies based at multiple locations. While a few international comparative studies exist that feature teacher and student interviews or classroom observations and provide some methodological insights, they focus rather on developing an overarching systematic (Bråten 2013) or discuss methodological issues only briefly (Knauth et al. 2008, 369–374). Furthermore, all these studies mostly remain on a national level of comparison and do not include regional contextual influences as part of the research process. In Bråten’s groundbreaking study, the ‘supranational’, ‘national’ and ‘subnational’ are identified as ‘three dimensions’ relevant to international comparisons in RE (2013, 44–47). While the ‘subnational dimension’ offers a suitable place to address questions of regionality in comparative RE, this is arguably the least developed of the three dimensions. Supranational influences are discussed concerning their influence on religion and education (47–52) and empirical data from teacher and student interviews is interpreted considering ‘national imaginaries’ (115–117) even though there is an awareness of local adjustments by the interviewed teachers (148–149). It seems that more work on the subnational – or regional – differentiation of RE would complement the previous work on comparative RE.

A focus on regionality finally also aligns with a larger trend in general comparative educational research towards a stronger focus on contextual differences. Cowen (2009) describes the identification of ‘unit ideas’ of comparison as a central problem for comparative education. While comparative education has made steady advances in terms of its techniques in comparison and data analysis, the formative effect of how the ‘units’ of comparison are conceptualised – most often as nation states or in a global/local framework – has often remained unnoticed. A diversification of the types of ‘unit ideas’ can lead to a more nuanced understanding of the realities of education as each ‘unit idea’ constrains and enables research in a different way. We argue that a focus on regionality and in-depth qualitative research can enrich the field of comparative RE in such a way.

This view is complemented by Powell’s (2020) observation that comparative education is increasingly being reduced to superficial analyses that lack deep contextual and historical knowledge. In his account, interest in comparative research is often fuelled by competition, which leads to reductionism as it is focused on single measures of accomplishment. Collaboration, on the other hand, can redeem this problem at least partly as it provides opportunities for ‘enhancing the potential for discovery’ (Powell 2020, 57) by integrating more expertise and awareness for contextual differences. It also allows for more complex research questions, higher degrees of critical reflection through multiple perspectives, overcoming cultural and disciplinary barrier as well as increased access to research networks and disciplinary communities (Powell 2020, 70–71). Thanks to higher mobility, better second language skills and online-communication tools, collaboration is now more feasible. However, it also produces new methodological challenges: ‘To coordinate complex comparative research projects requires organisational support, intercultural sensitivity, and tenacity’ (Powell 2020, 72). We view the MORE project as an attempt to advance such collaborations in comparative RE, which also faces the risk of reductionist accounts, especially when the analysis is focused solely on competing binary oppositions such as denominational or non-denominational RE. As Powell argues, the collaborative research mode entails challenges regarding the setup of research projects, which is why the following section will outline the principles and organisational decisions within the project in more detail.

### Study design: factoring in regional variability

From a comparative perspective, the task is to find a study design that is open for, and attentive to, regional characteristics, on the one hand, while still reaching a degree of methodological standardisation that allows for comparisons, on the other hand. With this section on the study design, we aim to show how different elements of the MORE project were organised in a way to meet this challenge.

#### Regional teams

Fundamental to the MORE-project is that every regional variant is researched by local researchers with expert knowledge of RE in that region. The project consists of teams of researchers with experience in teacher education and the internal debates within the regional variant at each of the participating universities (Zurich, Potsdam, Vienna and for Greater Stuttgart: Tuebingen). Even though the logistical lead of the project is at the University of Vienna, the MORE project is driven by a non-hierarchical, collaborative effort. This is important to prevent the perspective of one model or region to dominate the ways in which the other variants are researched. Instead, each team is responsible for data collection and analysis at its regional site.

#### Semi-structured guidelines

To balance this regional set-up of the research teams, the interview guidelines were developed by the whole team collaboratively and tested at different regional sites so that, finally, the same guidelines were used at each region. However, at the same time, the guidelines needed to be flexible enough so that they can be adapted to regional circumstances and to ensure that the interviewers could react appropriately to the answers of the teachers and pupils.

This means that regional or model-specific terminology was integrated wherever it would ensure a better understanding of the research questions. In the case of Zurich, the guideline had to be translated into Swiss German, and all but one of the interviews were conducted and transcribed in that language. This contextual adaptation also proved necessary to ensure a high quality of the interviews, as teachers and pupils participated more openly in Swiss German. In other cases, formulations concerning didactical concepts such as competencies had to be adapted.

More importantly, though, the guidelines were aimed at a concrete topic: didactic decisions on how to teach the topic of ‘Islam’ on the side of the teachers and the experience of being taught the topic of Islam on the side of the pupils. This means that despite the contextual differences, the interviews shared a similar point of reference. Also, teachers were not asked about abstract principles discussed in the academic debate on models of RE such as teaching about or from religion, but about their practical decisions concerning this particular topic. The guidelines include questions on the teachers’ aims, the content of their lessons, the methods they employ and the media they use. Also, they were asked about how they view the topic of ‘Islam’ themselves and their respective trainings. For the pupil-interviews, the guidelines included questions on what they had learnt in the lessons on ‘Islam’ and what kind of lessons they enjoy.

To ensure flexibility while also maximising standardisation, the teacher guidelines featured additional follow-up question that the interviewers could use to deepen the conversations. The pupil interviews started with the pupils developing a mind-map on the topic of ‘Islam’.

#### Sampling

The sampling process also had to balance standardisation and openness for regional specifics. This meant defining sampling criteria that applied to all four regions and ensuring a balanced sample in regard to gender, age and degree of urbanisation of the schools involved. However, at each region, particularities were also taken account of. For example, in Potsdam, there is a relatively high number of teachers who were trained for other subjects than ‘L-E-R’ but teach it nonetheless. In Stuttgart, there are both state-employed teachers and teachers employed by the Church, and in Zurich, there are teachers who have already taught in the model preceding RKE, which was called ‘Confessionally-cooperative RE’. It was the aim to include teachers from all these regional subgroups in order to enrich the sample in each region. The aim was not to reach representativeness in the same way as in large quantitative studies, but to include as many different perspectives from within one region as possible.

#### Data collection

For data collection again the principle that each regional team is responsible for its own data collection was applied. At the same time, some overarching preconditions were formulated. As mentioned above, the data consists of individual interviews with teachers, group interviews with three to five pupils of the teachers interviewed and an observation of one lesson by these teachers on the topic of ‘Islam’. The classroom observations were carried out by two researchers, each writing a protocol of the lesson. Additionally, basic information about all of the interviewees was gathered via questionnaires so that the demographic composition of the sample was recorded.

The decision to integrate multiple perspectives aimed at achieving an ‘ecological validity’ in which the views and actions of relevant actors in the classroom could be cross-checked with each other. It allows to investigate whether the pupils perceive the lessons in accordance with what the teachers say about them and what the researchers observed in the classrooms. Furthermore, the combination of interviews and observations meant that the interviews were always conducted in close proximity in time to the actual treatments of the topic of ‘Islam’ in the classroom.

However, the data collection was also affected by other contextual factors. For example, as the Covid-pandemic affected schools much more in Germany than in Switzerland, schools and teachers were more hesitant to participate in Germany than in Switzerland.

#### Data analysis

In the case of the data analysis, there was a twofold challenge of integration. Firstly, at each region, the results of the teacher and pupil interviews as well as the observations needed to be related to each other to achieve the ‘ecological validity’ they were meant to ensure. Secondly, the data needed to be analysed in a way that would enable comparisons between the regions.

The interviews were inductively analysed according to a method called ‘Qualitative content analysis’ (Mayring 2022) that is widely used in German-speaking countries. Using such an established method meant that at all study sites, researchers would share a basic understanding of the method from the beginning. As a relative of Grounded Theory, this method is based on inductively analysing the data by developing codes and categories. However, it differs from Grounded Theory in terms of the degree of formalisation for the individual steps in the analysis, leading to a system of categories that order and describe the data. This higher degree of rule-boundedness was helpful for the collaborative nature of the MORE project. While the regional teams each analysed their own data, still an integrated category system was developed and shared via the Cloud-function of the data-analysis software ‘MaxQDA’. This made it possible that during the process of coding the interviews, each person coding was notified as soon as a new category emerged so that they could include this category in their analysis as well. This was the case, for example, concerning a main category in the analysis of the teacher-interviews called *teachers’ conceptions of their pupils*. It soon became obvious that teachers in all regions talked similarly about their pupils regarding, for example, their consumption of (social) media and its negative influence on their perception of Islam. The other main categories extend from *didactics* to the *self-reflection of the teachers*, *framework conditions* and *model- and subject-related observations*. These main categories are differentiated into more than 50 sub-categories.

Regularly, the preliminary category system was discussed by the whole team to identify new categories, work on the definition of categories or find exemplary statements for each category. The result is a shared inductively developed category-system that is anchored in the data of all four regions and that integrates results from the analysis of both sets of interviews and the observation-protocols. This shared category-system also ensures that results are presented with similar terminology as it defines the categories and thus the key terms for the presentation and discussion of results.

#### Presentation and discussion of results

Because of the shared category-system, the results from each region can be described in parallelly structured case studies for each of the four regions. These case studies on the teaching the topic of ‘Islam’ as described by teachers, perceived by pupils and carried out in the classroom both stand on their own and form the basis of an in-depth comparison between the regions. Based on the case studies, commonalities and differences between regions that follow the same or different models of RE can be identified by comparing individual chapters of the case studies, for example, concerning the teachers’ goals or their preferred content.

## Conclusions and outlook

The analysis of the interviews and observations is still in progress, so that no final results can be discussed here. The project is scheduled to be completed in 2025 and two monographs as well as articles are planned to be published to present the final results. However, already based on the process of developing and contextualising the research designs, it becomes clear that regionality is highly important for a differentiated international comparison of RE. In addition to cultural, disciplinary and language-related differences, regional contextual factors that affected the design and organisation of the study include but are not limited to differences in teacher education and their (non-)religious affiliation, classroom sizes and heterogeneity of pupils, curricula as well as subject-specific requirements regarding its compound nature or the positioning of RE classes. While some of these factors align with the question of denominational vs. non-denominational RE, others do not and point to the necessity of addressing the full complexity of regional contexts in RE research, instead of reducing it to potentially simplified accounts of binary oppositions.

Considering recent advances to increase international knowledge transfer (Berglund et al. 2023) and to facilitate transnational exchanges in religious education as a field of research (Käbisch 2019), further exploring the effects of regions on the teaching of RE calls for the development of study designs which can integrate research in a collaborative mode across contextual and model-related borders. To this end, the MORE project’s design can potentially be adapted to research on other regions in the future. It, thus, provides a useful tool for a nuanced representation of the empirical reality of RE.

## Notes

1. We use the term ‘religion-related education’ to include all the (non-)denominational subjects that address religion within school teaching, which are sometimes referred to as ‘religious education’ or as ‘religion education’. In the following, it is abbreviated to RE.

2. The MORE project comprises four local teams with the following present team members: Potsdam (Stephanie von Steinsdorff, Marie-Luise Raters), Tübingen (Benjamin Ahme, Friedrich Schweitzer), Vienna (Claire Weiß, Sabine Hermisson, Martin Rothgangel) and Zurich (Rafaela Estermann, Thomas Schlag). The article is based on the collaborative research of the entire MORE team.

3. In German ‘Lebensgestaltung – Ethik – Religionskunde’.
